# The application of HER2 and CD47 CAR-macrophage in ovarian cancer

**DOI:** 10.1186/s12967-023-04479-8

**Published:** 2023-09-22

**Authors:** Yizhao Chen, Xiangling Zhu, Hanze Liu, Cunzhi Wang, Yu Chen, Huihui Wang, Yilong Fang, Xuming Wu, Yuting Xu, Chunhua Li, Xinyue Lv, Jinghua Huang, Xintong Han, Ruilin Li, Wenming Hong, Zhiying Yu, Wei Wei, Jiajie Tu

**Affiliations:** 1 Institute of Clinical Pharmacology, Key Laboratory of Anti-Inflammatory and Immune Medicine, Anhui Collaborative Innovation Center of Anti-Inflammatory and Immune Medicine, Anhui Medical University, Ministry of Education, #81 Meishan Road, Shushan District, Hefei, China; 2 Department of Neurosurgery, First Affiliated Hospital of Anhui Medical University, #218 Jixi Road, Shushan District, Hefei, China; 3 Department of Gynecology, Health Science Center, The First Affiliated Hospital of Shenzhen University, #3002 Sungangxi Road, Futian District, Shenzhen, China; 4 Shenzhen Second People’s Hospital, #3002 Sungangxi Road, Futian District, Shenzhen, China; 5 Department of Pharmacy, The Third Affiliated Hospital of Anhui Medical University, Hefei First People’s Hospital, Hefei, China

**Keywords:** Chimeric antigen receptor, Macrophages, T cell, HER2, CD47, Immunotherapy

## Abstract

**Background:**

The chimeric antigen receptor (CAR)-T therapy has a limited therapeutic effect on solid tumors owing to the limited CAR-T cell infiltration into solid tumors and the inactivation of CAR-T cells by the immunosuppressive tumor microenvironment. Macrophage is an important component of the innate and adaptive immunity, and its unique phagocytic function has been explored to construct CAR macrophages (CAR-Ms) against solid tumors. This study aimed to investigate the therapeutic application of CAR-Ms in ovarian cancer.

**Methods:**

In this study, we constructed novel CAR structures, which consisted of humanized anti-HER2 or CD47 scFv, CD8 hinge region and transmembrane domains, as well as the 4-1BB and CD3ζ intracellular domains. We examined the phagocytosis of HER2 CAR-M and CD47 CAR-M on ovarian cancer cells and the promotion of adaptive immunity. Two syngeneic tumor models were used to estimate the in vivo antitumor activity of HER2 CAR-M and CD47 CAR-M.

**Results:**

We constructed CAR-Ms targeting HER2 and CD47 and verified their phagocytic ability to ovarian cancer cells in vivo and in vitro. The constructed CAR-Ms showed antigen-specific phagocytosis of ovarian cancer cells in vitro and could activate CD8^+^ cytotoxic T lymphocyte (CTL) to secrete various anti-tumor factors. For the in vivo model, mice with human-like immune systems were used. We found that CAR-Ms enhanced CD8^+^ T cell activation, affected tumor-associated macrophage (TAM) phenotype, and led to tumor regression.

**Conclusions:**

We demonstrated the inhibition effect of our constructed novel HER2 CAR-M and CD47 CAR-M on target antigen-positive ovarian cancer in vitro and in vivo, and preliminarily verified that this inhibitory effect is due to phagocytosis, promotion of adaptive immunity and effect on tumor microenvironment.

**Supplementary Information:**

The online version contains supplementary material available at 10.1186/s12967-023-04479-8.

## Introduction

Chimeric antigen receptor (CAR) is a synthetic receptor. Its structure includes a single chain variable fragment or antigen recognition binding domain, a transmembrane domain providing scaffold and signal transduction, and costimulatory domain from the T-cell receptor (TCR), stimulating T-cell activation [1]. CAR can combine with cancer cell surface antigen to induce and enhance the killing effect of T cells on cancer cells [2]. In current research and clinical trials, CAR-T cell therapy has shown excellent therapeutic effects in hematological malignancies [3–5]. However, the efficacy of CAR-T cell therapy for solid tumors is not significant because the tumor microenvironment inhibits the activity of CAR-T cells, and the CAR-T cells have difficulty infiltrating the solid tumors [6]. Although researchers have tried to enhance the efficacy of CAR-T cell therapy for solid tumors using various methods, such as deleting inhibitory receptors, adding accessory genes in the structure of CAR, and combining them with other therapies, no breakthrough has been made so far [7–9].

Macrophages are important factors of the innate immunity, playing key roles in inflammation and host defense [10]. Macrophages majorly infiltrate solid tumors. Macrophages can phagocytize tumor cells, but cancer cells can express a “don’t eat me” signal to inhibit this phagocytosis. Moreover, under the influence of tumor microenvironment (TME), these macrophages are polarized into M2 anti-inflammatory macrophages, which play an important role in the development and metastasis of tumors [11]. Considering the unique phagocytic function of macrophages and their ability to infiltrate solid tumors, researchers have attempted to construct CAR macrophages to fight solid tumors. Klichinsky et al. first constructed CAR-Ms to treat solid tumors. They confirmed the targeted phagocytosis of tumor cells and activation of adaptive immunity by CAR-M in human immune system mice [12]. To obtain a stable source of CAR-Ms, Zhang et al. derived CAR-M cells from induced multifunctional stem cells [13]. Furthermore, Mikyung Kang et al. used nanomaterial-mediated gene transfer to program macrophages expressing CAR and anti-tumor M1 phenotypes in animals and confirmed their effectiveness [14]. Recently, Fu et al. constructed CAR-Ms containing the MERTK cytoplasmic domain to phagocytize SARS-COV-2 virus particles and found that there was no pro-inflammatory reaction in this process; this is another research direction of CAR-M [15]. In addition to their application in tumor immunity, suggesting that the functions of macrophages can be enhanced through the CAR strategy.

The HER2 gene has been shown to be strongly related to the development of breast cancer and amplified in a variety of solid tumors, and HER2 status is associated with recurrence and metastasis of ovarian cancer and it is only expressed in very few normal tissues [16]. Therefore, HER2 is an ideal target for solid tumor therapy. On the other hand, CD47 is usually expressed on senescent cells and is abundantly expressed on the surface of various malignant tumor cells [17]. Elevated CD47 expression is associated with low survival in these cancer patients [18]. The CD47-SIRPα signaling pathway is thought to be a key mechanism by which cancer cells evade innate immune surveillance [18]. These findings make CD47 a valuable target for cancer immunization.

In this study, we constructed CAR-Ms that target HER2 and CD47, respectively and investigated their ability to phagocytize ovarian cancer cells and activate adaptive immunity in vivo and in vitro. The constructed CAR-Ms showed antigen-dependent phagocytosis of ovarian cancer cells in vitro and stimulated the activation of CD8^+^ T cells to secrete various cytokines to kill cancer cells. CAR-Ms can inhibit xenotransplantation of SKOV3 cells in vivo, enhance CD8^+^ T cell response in syngeneic tumor-bearing NCG mice with Hu-PBMC model, affect macrophage phenotype in TAM, and lead to tumor regression.

## Materials and methods

### Cells

Two human ovarian cancer cell lines (SKOV3 and A2780) and a human monocyte line THP-1 were purchased from Procell Life Science & Technology. SKOV3 cells were cultured in McCoy’s 5 A medium (Biological Industries) containing with 10% fetal bovine serum (FBS, Biological Industries) and 1% penicillin/streptomycin (Beyotime). A2780 cells were cultured in RPMI 1640 (Biologial Industries), containing with 10% FBS and 1% penicillin/streptomycin. THP-1 cells were maintained in RPMI 1640 medium containing with 10% FBS. All cells were cultured in an incubator with 5% CO_2_ at 37 °C.

### CAR macrophage cells production

THP-1 cells were stimulated to differentiate into human macrophages using 100 ng/ml PMA (P1585-1MG, Sigma), and the stimuli were evaluated using CD11b and CD68 expression for subsequent experiments. CAR adenovirus was purchased from the OBiO Technology Company Limited (Shanghai, China). CAR THP-1 cells targeting CD47 and HER2 and vehicle control cells were prepared by adenovirus infection of PMA-stimulated THP-1 cells according to the manufacturer’s instructions. After infection, CAR THP-1 cells and vehicle control cells were cultured in RPMI1640 supplemented with 10% FBS and 100 ng/ml recombinant human granulocyte monocyte colony-stimulating factor (GM-CSF, 300-03, PeproTech). After three days, the cells were analyzed by flow cytometry for Green fluorescent protein (GFP) expression to determine CAR expression.

### FACS-based phagocytosis assay

The human ovarian cancer cell lines SKOV3 and A2780 (1 × 10^5^) were labeled with the CellTracker™ Red CMTPX (C34552, Invitrogen) and co-cultured with CAR THP-1 cells expressing GFP fluorescent protein or a vehicle control (1 × 10^5^). After 4 h, Cells were harvested and cell suspensions were pelleted and washed with 0.1% BSA in phosphate-buffered saline (PBS, BaseIMedia), and analyzed by flow cytometry.

### Evaluation of macrophage-dependent activation of CD8^+^ T cells

The human ovarian cancer cell lines SKOV3 and A2780 (5 × 10^5^) were co-cultured with CAR THP-1 cells or vehicle control (5 × 10^5^) and incubated for 4 h (at 37 °C and 5% CO_2_). Whole blood from healthy blood donors was diluted 1:1 in PBS and layered in human lymphocyte isolation medium. Peripheral blood mononuclear cells (PBMC) were separated by density gradient centrifugation in a 50 ml tube. CD3^+^ T cells in PBMC were separated using CD3 magnetic beads (130-097-043, Miltenyi Biotec). Purified CD3 ^+^ T cells (5 × 10^5^) were immediately added to the co-culture system of tumor cells and CAR THP-1 or vehicle control and cultured for 48 h at 37 °C and 5% carbon dioxide incubator. T cells were harvested, resuspended in PBS, and labeled with anti-human CD8 (344,747, BioLengend), anti-human IFN-γ (2,331,102, Invitrogen), Fasl (306,421, BioLegend) and CD40L (310,824, BioLegend) antibodies at 4 ℃ for 30 min. After centrifugation at 370 *g* for 5 min, 200 µl PBS was used to resuspended cells and detected by flow cytometry. CAR-M cells were harvested, resuspended in PBS, and labeled with anti-humanCD40 (40,334,308, BioLegend) antibodies at 4 ℃ for 30 min. After centrifugation at 370 *g* for 5 min, 200 µl PBS was used to resuspended cells and detected by flow cytometry.

### Cytokine analysis

Cytokine analysis was carried out on the supernatants extracted from the cultures by ELISA using different ELISA kits according to the manufacturer’s instructions. The human cytokine IL-2 (ELH-IL-2, RayBio), INF-γ (ELH-IFNg, RayBio), TNF-α (ELH-TNFα, RayBio), GzmB (ELH-GZMB, RayBio), TNF-β (EK17004, SABbiotech) and The Perforin 1 (EK16250, SABbiotech) ELISA kits were used. The results were read on a multifunctional microplate reader (Tecan, Infinite M1000 PRO).

### Xenograft tumor model in nude mice

BALB/c nude mice (female, 18 ± 4 g, five weeks old) were purchased from the Animal Model Research Center. The animals were kept in the SPF Animal Laboratory of the Institute of Clinical Pharmacology, Anhui Medical University (Hefei, China), and fed ad libitum. All experiments were approved by the Animal Experimental Ethics Review Committee of the Anhui Medical University (PZ-2022-004). Schemes of the xenograft models used are shown in detail in the first panel of each figure. Nude mice were divided into three groups: SKOV3 + untransfected THP-1 cells, SKOV3 + vehicle control, and SKOV3 + HER2 CAR THP-1 cells. Each mouse was transplanted with a mixture of SKOV3 cells (1 × 10^6^) and THP-1 cells (3 × 10^6^). A subcutaneous injection was performed under the armpit of each nude mice, and all cells were mixed evenly in 100 µl PBS before injection. Tumor volume was calculated using the following formula: length × width^2^ × 0.5.

### Syngeneic tumor-bearing NCG mice

NOD/ShiLtJGpt-Prkdc^em26Cd52^Il2rg^em26Cd22^/Gpt (NCG) mice were purchased from GemPharmatech Co., Ltd. (Nanjing, China). The animals were kept in the SPF Animal Laboratory of the Institute of Clinical Pharmacology, Anhui Medical University (Hefei, China). All experiments were approved by the Animal Experimental Ethics Review Committee of the Institute of Clinical Pharmacology, Anhui Medical University (PZ-2022-004). SKOV3 cells (2 × 10^6^) were resuspended in 100 µl PBS, mixed well and injected subcutaneously into the armpits of each NCG mouse. The tumor volumes of animal models were calculated according to the following formula: length × width^2^ × 0.5.

### Establishment of Hu-PBMC models

Human whole peripheral blood mononuclear cells were isolated using Lymphoprep (Milestone Biotechnologies). NCG mice that had formed tumor bodies in the axilla were injected with 1 × 10^7^ hu-PBMCs via the tail vein. At days 14–21, the tail vein was collected to detect the proportion of hCD45^+^ cells in PBMC by flow cytometry, and more than 25% could be considered a successful model construction. Body weight was recorded every three days after the tail vein hu-PBMC injection.

### Immunohistochemistry

The experimental process of Immunohistochemistry was conducted by following a standard protocol. The primary antibodies were included: anti-proliferating cell nuclear antigen (PCNA) (ab92552, Abcam), MHC II (ab180779, Abcam), CD163 (ab182422, Abcam), IFN-γ (ab218426, Abcam), HER2 (ab134182, Abcam), CD47 (ab218810, Abcam), VEGF (ab1316, Abcam).

### Western blotting

The experimental process of Western blotting was conducted by following a standard protocol. The primary antibodies used were PCNA (ab92552, Abcam), MHC II (ab180779, Abcam), CD163 (ab182422, Abcam), and CD8α (Cell Signaling Technology, 98941T).

### Flow cytometry

Tumor tissues taken from nude mice successfully established models and NCG mice treated with CAR-M were minced and digested into single cells. The cells were resuspended in 100 µl PBS and incubated with the indicated antibodies at 4 °C for 40 min. Anti-human CD68 (2,172,621, eBioscience), anti-human CD86 (305,425, BioLegend), anti-human CD206 (130-100-230, Miltenyi Biotec), anti-human CD3 (300,308, BioLegend), anti-human CD8 (344,747, BioLegend), anti-human Ki67 (350,514, BioLegend) anti-mouse CD47 (127,505, BioLegend) antibodies were used. The cells were washed twice and analysis using a flow cytometer (Beckman CytoFLEX). The data were analyzed with CytExpert analysis software (Beckman).

### Statistical analysis

Student’s t-test, one-way analysis of variance (ANOVA), and Kaplan–Meier survival curves were used for statistical analysis by using GraphPad Prism 8.0. All experiments were performed independently, at least in triplicate, and the data are expressed as mean ± standard error (SEM). Significance was set at P < 0.05 was considered to indicate a significant difference.

## Results

### The construction of HER2 CAR-M and CD47 CAR-M and their phagocytosis ability in vitro

**Fig. 1:**
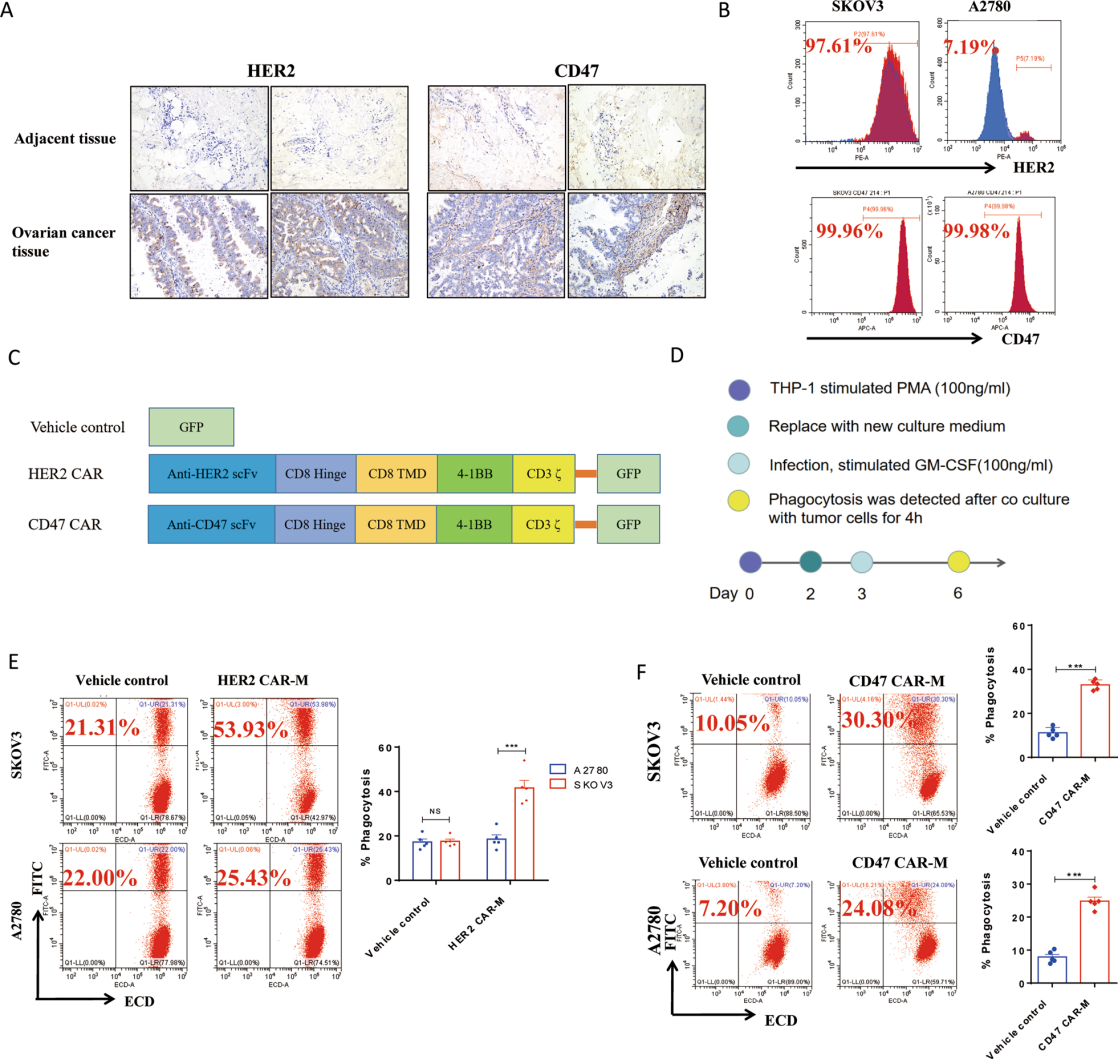
The construction of HER2 and CD47 CAR-M and phagocytosis in vitro. **A** The expression and localization of HER2 and CD47 in the tumor tissues of patient with ovarian cancer. **B** The expression of HER2 and CD47 in two ovarian cancer cell lines SKOV3 and A2780. **C** The chimeric antigen receptor structure used in this study includes an anti-CD47/HER2 scFv segment, a hinge region and transmembrane domain from CD8, 4-1BB and CD3ζas intracellular domains, and a GFP fluorescent tag attached. **D** The experimental flow of constructing CAR-M and subsequent in vitro experiments. **E** The phagocytosis of HER2 CAR-M on HER2^lo^ SKOV3 and HER2^hi^ A2780 (n = 5). **F** The phagocytic effect of CD47 CAR-M on CD47^hi^ SKOV3 and A2780 (n = 5). *ns* not significant; ^**^ P < 0.01; ^***^ P < 0.001

We first examined the expression and localization of two oncogenes, HER2 and CD47, in tumor tissues of patients with ovarian cancer. Immunohistochemistry staining showed significantly elevated expression of HER2 and CD47 in tumor tissues compared with adjacent tissues (Fig. 1A). We designed HER2-targeting and CD47-targeting CAR structures and infected PMA-differentiated THP-1 macrophages with adenoviral vectors to generate HER2 CAR-M and CD47 CAR-M in vitro (Fig. 1C and Additional file 1: Fig. S1A). Adenoviral vectors can obtain high infection efficiency (Additional file 1: Fig. S1B). Figure 1D shows the experimental flow for constructing the CAR-Ms and subsequent in vitro experiments. Flow cytometry showed that two ovarian cancer cell lines SKOV3 and A2780 expressed high and low HER2, respectively, while CD47 was highly expressed in both cell lines (Fig. 1B). These two ovarian cancer cell lines were co-cultured with CAR-M to evaluate the targeted phagocytosis of CAR-M against ovarian cancer. Flow cytometry showed that the phagocytosis of the HER2 low-expressing (HER2^low^) cell line A2780 by HER2 CAR-Ms was not significant, but the phagocytosis of HER2 high-expressing (HER2^hi^) SKOV3 cells was significant (Fig. 1E). Moreover, the phagocytic effect of CD47 CAR-Ms on both cell lines, SKOV3 and A2780, was high compared with that in the control group (Fig. 1F). The experimental results suggested that we successfully constructed HER2 CAR-Ms and CD47 CAR-Ms and verified the targeted phagocytosis effect on ovarian tumor cells in vitro (Additional file 1: Fig. S2).

### The effects of HER2 CAR-Ms and CD47 CAR-Ms on cytotoxic T lymphocyte

**Fig. 2:**
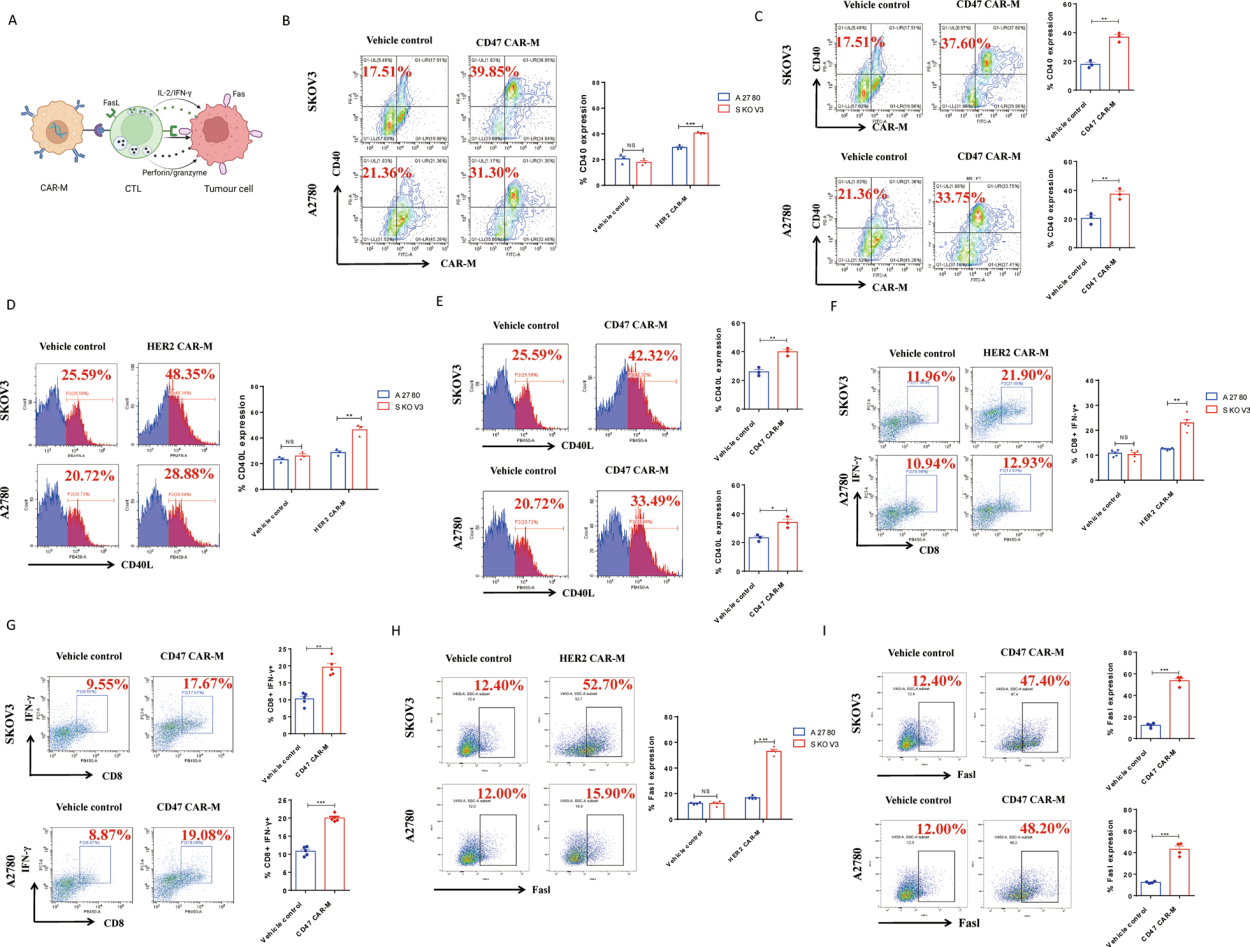
The effects of HER2 CAR-M and CD47 CAR-M on cytotoxic T lymphocyte. **A** The experimental flow to evaluate the effects of CAR-M on CTL in vitro. **B** Effect of CD3^+^ T cells on CD40 expression of HER2 CAR-M (n = 3). **C** Effect of CD3^+^ T cells on CD40 expression of CD47 CAR-M (n = 3). **D** Effect of HER2 CAR-M on CD40L expression in CD3^+^ T cells (n = 3). **E** Effect of CD47 CAR-M on CD40L expression in CD3^+^ T cells (n = 3). **F** The effects of HER2 CAR-M on the differentiation of CD3^+^ T cells into CTL (n = 4). **G** The effects of CD47 CAR-M on the differentiation of CD3^+^ T cells into CTL ()n = 4. **H** Effect of HER2 CAR-M on Fas/Fasl Signal Pathway of T Cells (n = 4). **I** Effect of CD47 CAR-M on Fas/Fasl Signal Pathway of T Cells (n = 4). *ns* not significant; ^*^ P < 0.05; ^**^ P < 0.01; ^***^ P < 0.001

**Fig. 3:**
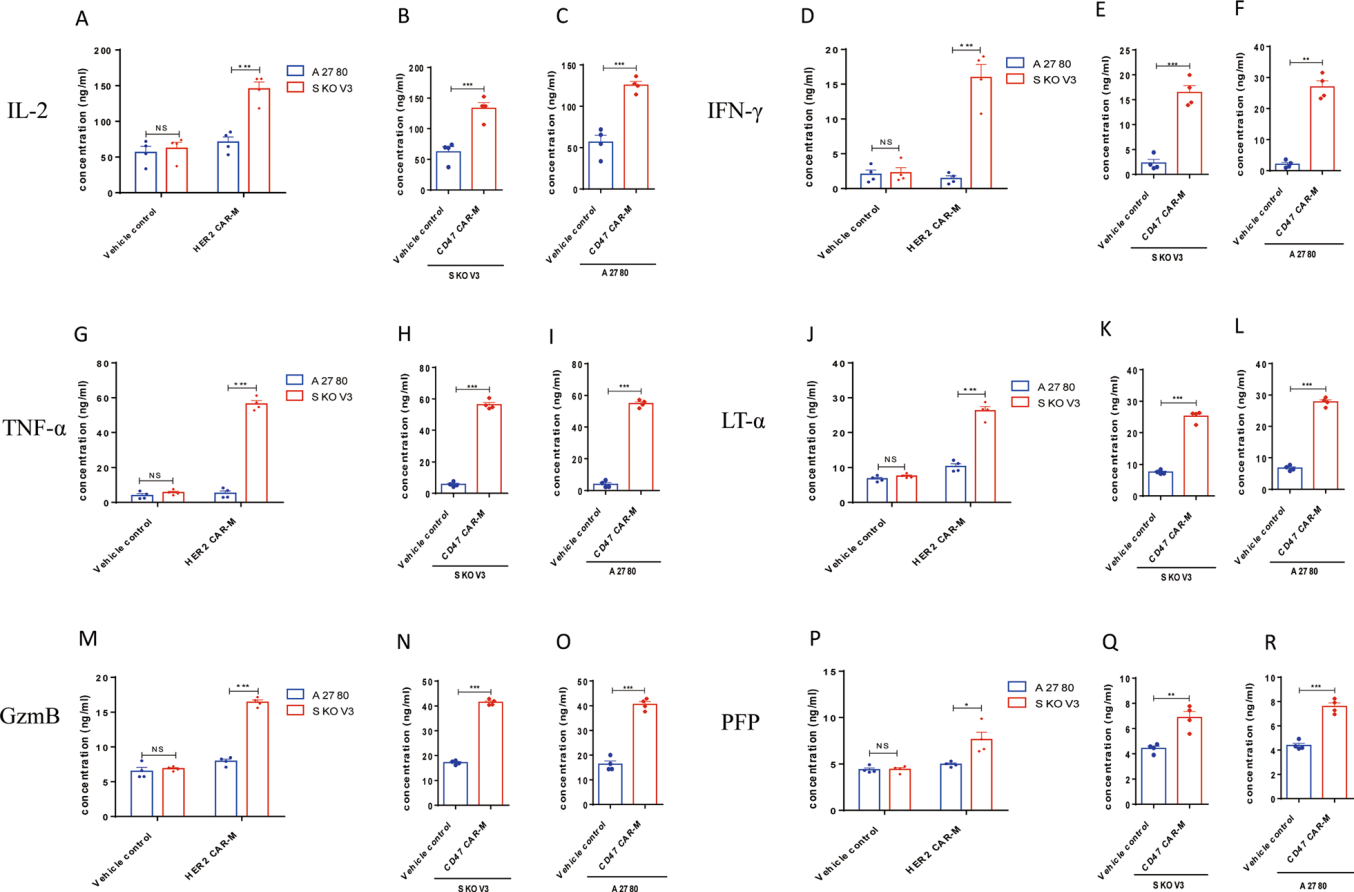
**A**–**R** ELISA results of CTL-secreted cytokines after activation by HER2 CAR-M and CD47 CAR-M, including IL-2, IFN-γ, TNF-α, LT-α, granulose and perforin (n = 4). *ns* not significant; ^*^P < 0.05; ^**^P < 0.01; ^***^P < 0.001

As antigen-presenting cells (APC), macrophages can promote the killing effect of T cells on tumor cells (Fig. 2A). Here, we first tested whether CAR-M, which engulfed tumor cells, could present degraded cell debris to T cells as tumor-associated antigen, thereby promoting the differentiation of T cells into cytotoxic T lymphocytes (CTLs). The flow cytometry results showed that HER2 CAR-Ms significantly promoted the differentiation of CD3^+^ T cells into CTLs (Fig. 2F) after engulfing the SKOV3 ovarian cancer cells with high HER2 expression compared with the control group. Similarly, CD47 CAR-M also significantly promoted the differentiation of CD3^+^ T cells into CTLs (Fig. 2G), after engulfing the two ovarian cells (SKOV3 and A2780) with high CD47 expression. Furthermore, we examined the expression of CD40 on the surface of CAR-M and CD40L on CD3^+^ T cells after co-culture, and the results of flow cytometry indicated a significant increase of CD40 and CD40L (Fig. 2B–E), suggesting an direct interaction between CAR-M and CD3^+^ T cells. One of the most important ways CTL plays its role in killing tumor cells is by secreting a variety of important cytokines, mainly via three pathways: cell lysis caused by perforin/granulose, tumor cell apoptosis caused by cytokines FasL/TNF-α/LT-α/FasL, and immunoregulatory effects induced by IFN-γ and IL-2. ELISA and flow cytometry results showed that the CTLs activated by HER2 CAR-Ms and CD47 CAR-Ms significantly expressed cytokines involved in the three signaling pathways mentioned above, including perforin/granulose, IL-2/TNF-α/LT-α/IFN-γ, and FasL (Figs. 2H, I,  3A–R).

### The applications of HER2 CAR-Ms in vivo

**Fig. 4:**
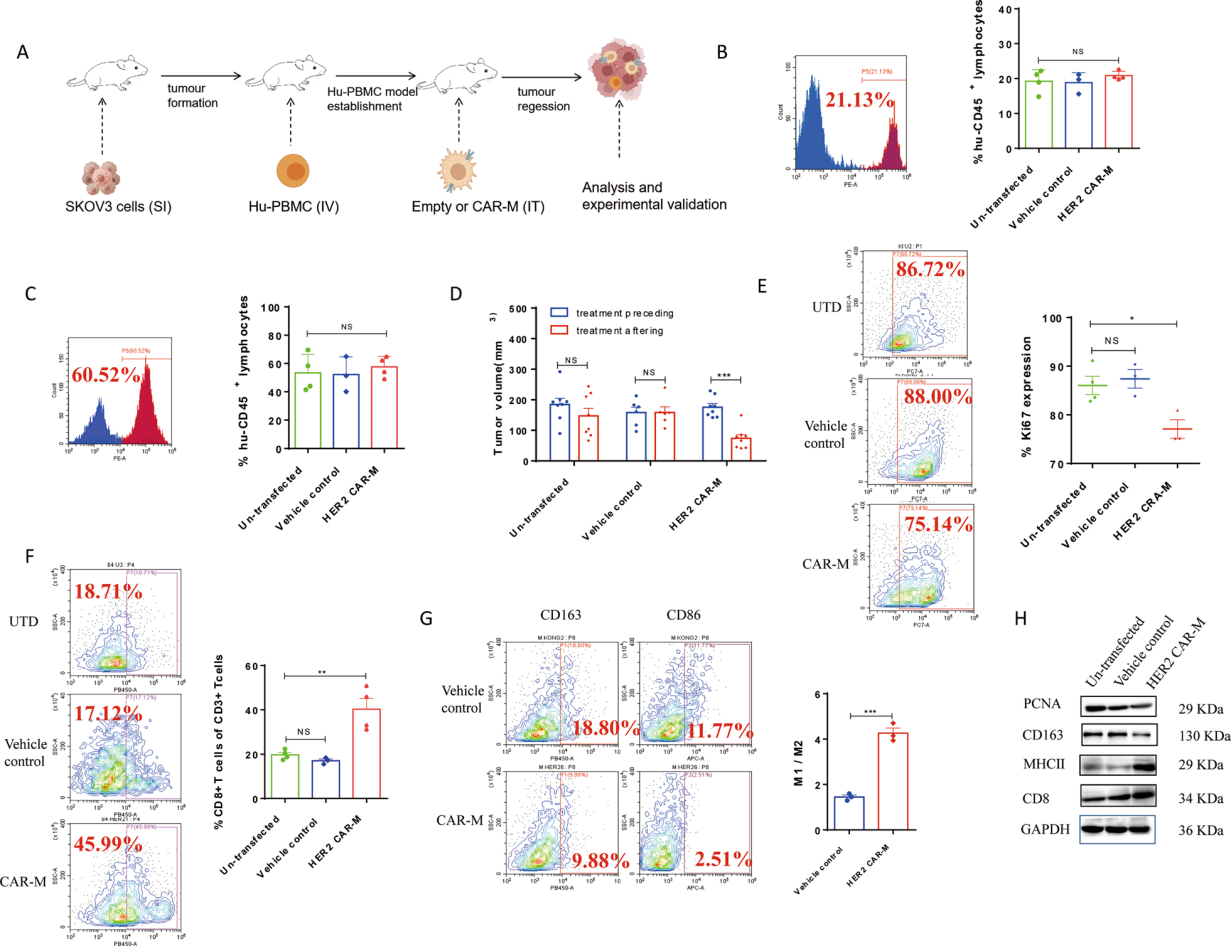
The applications of HER2 CAR-M in vivo. **A** The experimental flow to evaluate the therapeutic effects of HER2 CAR-M on tumor growth in vivo. **B** 14 days after the establishment of Hu-PBMC model, the proportion of hu-CD45^+^ cells in mouse PBMC (n = 3). **C** The percentage of hu-CD45^+^ cells in PBMC of mice after killing (n = 3). **D** The inhibitory effect of HER2 CAR-M on tumor growth of HER2^hi^ SKOV3 cell-formed tumors (n = 6). **E** Flow cytometry analysis of tumor proliferation marker Ki67 (CD11b^−^Ki67^+^) in tumor tissues (n = 3). **F** Flow cytometry analysis of CD8^+^CD3^*+*^ CTL (n = 3). **G** Flow cytometry results of ratio of M1/M2 in the tumor tissues (n = 3). **H** Western blot results of PCNA, MHCII, CD163 and CD8 expression in tumors (n = 3). *ns* not significant; ^*^P < 0.05; ^**^P < 0.01; ^***^P < 0.001

**Fig. 5:**
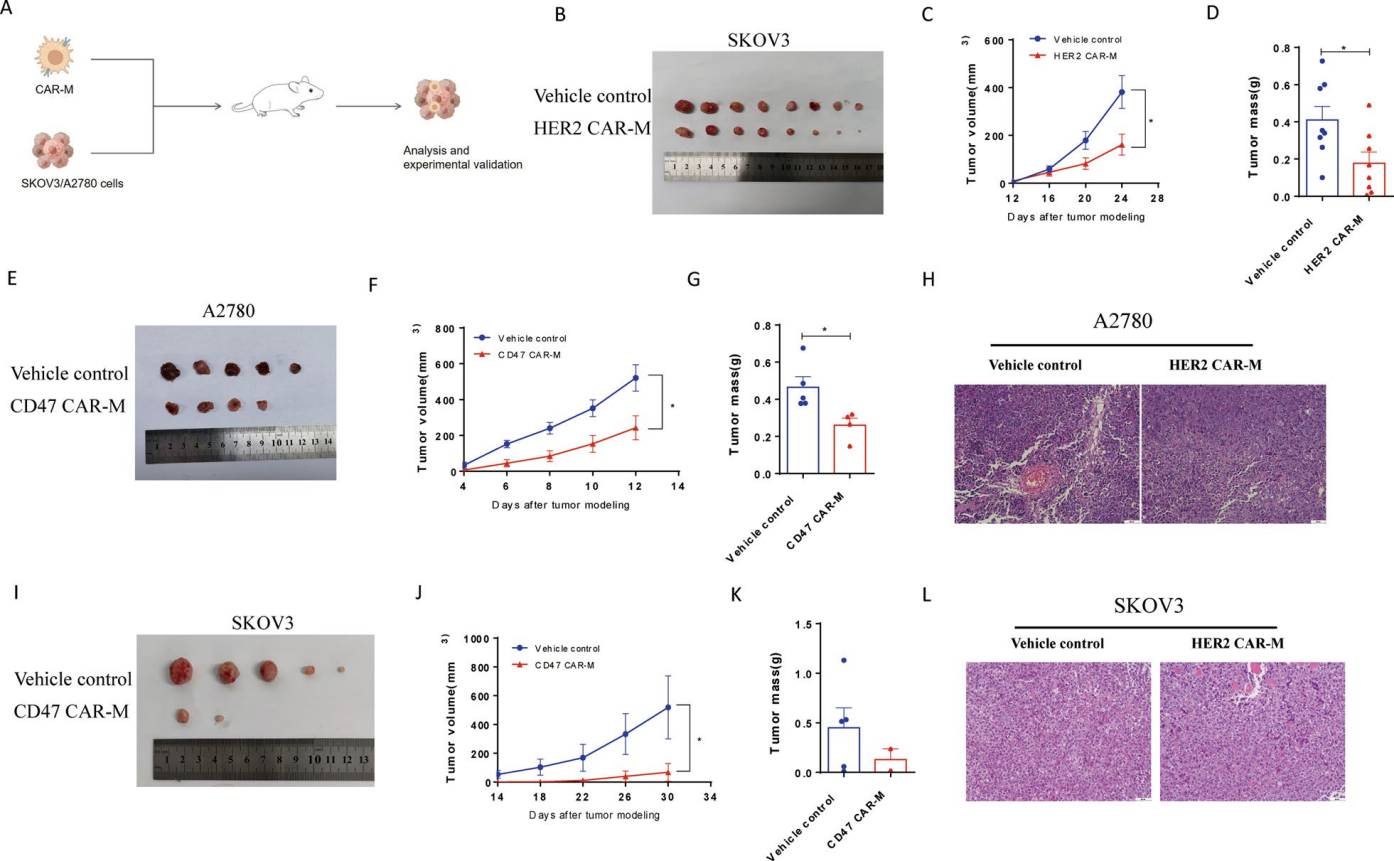
The applications of HER2 CAR-M and CD47 CAR-M in vivo*.* **A** To evaluate the experimental process of the effect of HER2 CAR-M or CD47 CAR-M on tumorigenicity and growth of tumors in vivo. **B**–**D** Effect of CD47 CAR-M on tumorigenicity and growth of HER2^hi^ SKOV3 cells (n = 8). **E**–**H** Effect of CD47 CAR-M on tumorigenicity and growth of CD47^hi^ A2780 cells (n = 5). **I**–**L** Effect of CD47 CAR-M on tumorigenicity and growth of CD47^hi^ SKOV3 cells (n = 5). *ns* not significant; ^*^P < 0.05; ^**^P < 0.01; ^***^P < 0.001

A humanized immune system mouse model was used in vivo to assess the inhibitory effect of HER2 CAR-Ms on tumor formation in the HER2^hi^ ovarian cancer cell line SKOV3 (Fig. 4A–C and and Additional file 1: Fig. S1D). Compared with the control group, HER2 CAR-Ms significantly inhibited the growth of SKOV3 cells (Fig. 4D). Consistent with this result, flow cytometry analysis showed that the proportion of the proliferation marker Ki67 (CD11b^−^Ki67^+^) was reduced in tumor tissues in the HER2 CAR-M group (Fig. 4E). In addition, the expression of PCNA in tumor tissue was also reduced (Fig. 4H). Interestingly, an increase in the proportion of CD8^+^CD3^*+*^ CTL in the HER2 CAR-M group was found from the flow cytometry results (Fig. 4F), suggesting that HER2 CAR-M could promote the differentiation of CTL in vivo, that is consistent with the in vitro results. At the same time the expression of CD8 in tumor tissues was also validated the FACS results (Fig. 4H). Moreover, we assessed the proportion of overall macrophage polarization marker M1/M2 in the tumor microenvironment. To exclude potential interference of the adenoviral vectors, we confirmed by flow cytometry that the adenoviral vector used did not have a direct effect on the polarization of HER2 CAR-M (Additional file 1: Fig. S1C). The flow cytometry results showed an induction in the ratio of M1/M2 macrophages in the tumor tissues in the HER2 CAR-M group compared with that in the control group (Fig. 4G), implying that the overall macrophages in the tumor microenvironment of the HER2 CAR-M treatment group were maintained in a tumor-suppressing M1 state. Western blot and IHC results also support this conclusion (Fig. 4H and Additional file 1: Fig. S3). Additionally, we also evaluated the effect of HER2 CAR-M on tumorigenicity and tumor growth of HER2^hi^ SKOV3 cells in a nude mouse model (Fig. 5A). Compared with the control group, HER2 CAR-M significantly inhibited the proliferation of HER2^hi^ SKOV3 cells (Fig. 5B–D and Additional file 1: Fig. S4A, D, E), suggesting that HER2 CAR-M could efficiently repress the growth of HER2^hi^ ovarian cancer cells-formed tumor. However, the M1 and M2 marker didn’t change in HER2 CAR-M treatment group when compared to vehicle control (Additional file 1: Fig. S4B, C, F). This is inconsistent with the conclusions drawn by the mouse model of the humanized immune system, and the differences in the two models suggest that the interaction between CAR-M and other immune cells may be responsible for its M1 polarization.

### The applications of CD47 CAR-Ms in vivo

To evaluate the effect of CD47 CAR-M on tumorigenicity and tumor growth of ovarian cancer cells, two CD47^hi^ ovarian cancer cell lines SKOV3 and A2780 were transplanted in nude mice subcutaneously (Fig. 5A). Compared with the control group, CD47 CAR-M significantly inhibited the growth of CD47^hi^ A2780 cells and SKOV3 cells-formed tumor (Fig. 5E–L and Additional file 1: Fig. S5). It is the first time to prove that CD47 CAR-M could efficiently suppress the CD47^hi^ ovarian cancer cells-formed tumor, which is worth further investigation for its potential application of CD47^hi^ ovarian cancer. In addition, the erythrocyte toxicity of CD47 CAR-M should be evaluated considering the high expression of CD47 on the erythrocyte surface. The in vivo toxicity of CD47 CAR-M were evaluated in WT mice, which showed a slight decrease in body weight and a decrease in the percentage of CD47^+^ cells in erythrocytes after administration of a therapeutic amount of CD47 CAR-M intravenously (Additional file 1: Fig. S6).

## Discussion

**Fig. 6:**
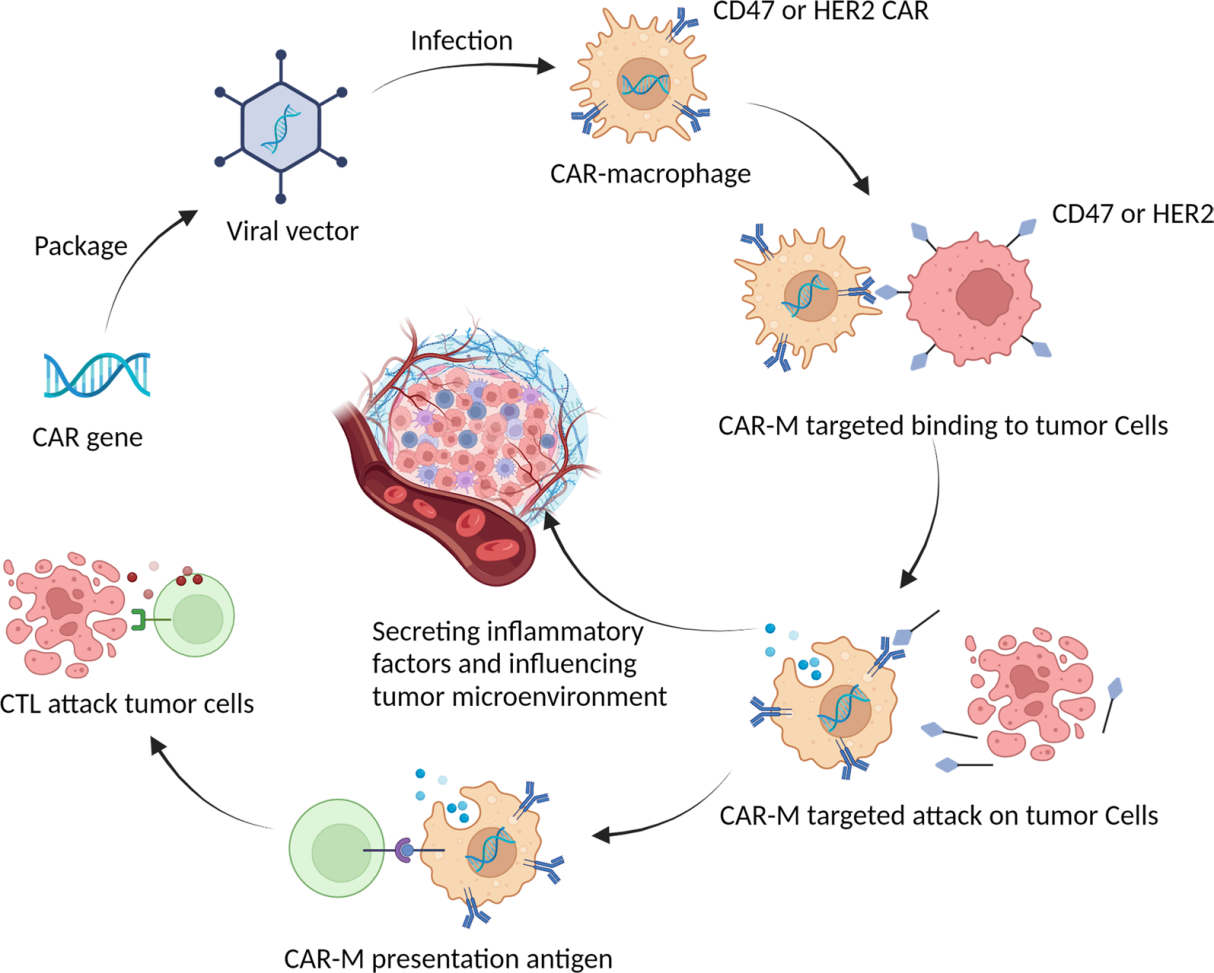
Outline diagram of the anti-tumor mechanism of HER2/CD47 CAR-M. The CAR gene was introduced into macrophages by viral transfection to obtain CAR-expressing CAR-M, HER2/CD47 CAR-M inhibits ovarian cancer growth and proliferation by phagocytosis both in vitro and in vivo, promoting adaptive immunity and influencing the tumor microenvironment (created with BioRender.com).

Among CAR-related therapies, CAR-M shows great potential against solid tumors. CAR-M has a unique phagocytic activity against tumors while promoting antigen presentation to T cells and enhancing the body’s adaptive immunity (Fig. 6). Macrophages are main immune regulatory cells and the most infiltrated immune cells in the TME [11]. Klichinsky et al., constructed an adenovirus vector Ad5F35 that induced the polarization of M2 macrophages to the M1 phenotype in the TME, which is consistent with our results. These findings suggest that CAR-M may enhance antitumor immunity by reducing the proportion of M2 macrophages in the TME and remodeling the anti-inflammatory status in TME. Moreover, the limited lifespan of macrophages indicates a low risk of non-tumor toxicity [19]. A prominent problem in CAR-T cell therapy is the cytokine storm caused by the activation and rapid proliferation of T-cells [20]. However, in the treatment of solid tumors, CAR-M may not cause a cytokine storm because CAR-M acts locally against the tumor. Moreover, previous studies showed that the infusion of CAR-M can lead to high levels of pro-inflammatory cytokines in tumors, while the level of pro-inflammatory cytokines in serum is low [21].

Nevertheless, there are still many problems to be overcome with CAR-M therapy. First, the number of macrophages is limited; because macrophages do not proliferate after infusion in vitro or in vivo, patients can only receive a limited number of CAR-M treatments, which may affect their efficacy [22]. CAR-M derived from induced multifunctional stem cells developed by Zhang et al. may provide a method to solve this problem [13]. Second, is the influence of the migration characteristics of macrophages in the body; most of the exogenous macrophages remain in the liver after injection, which is not conducive to the treatment of cancer [23]. Furthermore, there is the impact of the tumor microenvironment: Although CAR-M has achieved good efficacy in mouse models and can even reverse the polarization of macrophage M2 phenotype to M1 in TME, the actual tumor microenvironment in humans is more complex than that in animal models [24]. Owing to the high heterogeneity of tumor cells, the expression of target antigens may not be sufficient to trigger a series of anti-tumor effects mediated by CAR or make treated tumors develop into target antigen negative tumors through immune escape [25]. The achievement of CAR-M function relies on the targeted binding of CAR to the target antigen and the subsequent intracellular activation signaling [26]. One issue that must be considered is the effect of antigen expression levels on the surface of cancer cells on CAR-M activation. Although there have been no individual studies, similar problems in CAR-T therapy may give us some inspiration: in addition to simple recognition and binding of epitopes, the affinity of CAR’s antigen-binding domain and the target antigen fundamentally determines the function of CAR, and appropriate affinity is necessary to achieve the best therapeutic effect [9, 27]. This also provides an idea for the safe application of CAR-M, a moderate affinity of CAR-M may avoid unnecessary non-targeted toxicity and achieve better therapeutic results. Finally, in the study of Wang et al., a previously unrecognized mechanism was proposed: The absence of CAR may be an important reason for the failure of CAR-T, and this problem is also likely to appear in CAR-M [28].

The location of the CAR-binding epitope and the distance from the cellular surface also could affect antigen binding and cell activation of CAR-NK [29]. Therefore, the development of macrophage-specific CAR structures may enhance the targeted combination of CAR-M and tumor cells to improve efficacy. Moreover, CAR connecting Megf10 and FcRv intracellular domains can enhance the phagocytic activity of macrophages, and the assembly of phagocytic effector motifs can improve the whole cell phagocytic activity of CAR-M [30]. Fu et al., found that the MERTK domain can enhance the uptake of virus particles by macrophages without affecting the secretion of inflammatory cytokines [15]. These results suggest that the association of different intracellular domains may lead to great therapeutic benefits for CAR-M. Another promising direction for tumor treatment is combination therapy; the combination of engineered macrophages and some therapies has achieved remarkable results. Bian et al. revealed that macrophages in tumors resist to chemotherapy and other immunomodulatory methods using the negative regulator SIPRα. Utilizing SIRPα-KO cells proved the efficacy of radiotherapy combined with engineered macrophages [31]. Antibody-based immunotherapy can activate the phagocytic activity of macrophages and enhance adaptive immunity. Another direction of interest is the phagocytic checkpoints of innate immune cells, such as CD47, CD24, MHC-I, STC-1, GD2, SLAMF3/4, evade macrophage clearance via “don’t eat me” signaling [32, 33]. Among them, CD47 has been more intensively studied, and SIRPα is an essential ligand of CD47, which is mainly expressed in myeloid cells. The CD47-SIRPα axis blocks myosin-IIA accumulation, inhibits phagocytic prominence, and furthermore induces cross-presentation of APCs to initiate adaptive immunity [34]. Immunotherapies targeting CD47-SIRPα are in development, and our CD47 CAR-M is a meaningful attempt of this theme in cell therapy. Monoclonal antibodies against myeloid cell immune checkpoint CD47 enhance macrophage-mediated immune effects [35], and blocking the PD-1 signal pathway of T cell immune checkpoint has been proven to improve the phagocytic capacity of macrophages [36]. Generally, CAR-M may not only play the role of a tumor cell scavenger in the future, but its positive effect on adaptive immunity may also be more important in the combined application of some therapies.

### Supplementary information

**Additional file 1: Fig. S1.** Some characteristics of CAR-M obtained through adenovirus infection. **Fig. S2.** Image of CAR-M phagocytosis of ovarian cancer cell. **Fig. S3.** Immunohistochemistry results of mice tumor tissue after HER2 CAR-M treatment. **Fig. S4.** Immunohistochemistry results of SKOV3 cell and HER2 CAR-M tumor bearing mice. **Fig. S5.** Immunohistochemistry results of SKOV3/A2780 cell and CD47 CAR-M tumor bearing mice. **Fig. S6.** Preliminary evaluation of the in vivo safety of CD47 CAR-M.

## Data Availability

The data that support the findings of this study are available from the corresponding author upon reasonable request. Some data may not be made available because of privacy or ethical restrictions.

## Abbreviations

CAR: Chimeric antigen receptor
CAR-M: Chimeric antigen receptor macrophages
CTL: Cytotoxic T lymphocyte
FBS: Fetal bovine serum
GFP: Green fluorescent protein
PBMC: Peripheral blood mononuclear cells
PCNA: Pproliferating cell nuclear antigen
TAM: Tumor-associated macrophage
TCR: T-cell receptor
TME: Tumor microenvironment
