# Supplementary material for: The application of HER2 and CD47 CAR-macrophage in ovarian cancer
Source: J Transl Med. 2023 Sep 22;21:654. doi: 10.1186/s12967-023-04479-8 (PMC10517545; doi:10.1186/s12967-023-04479-8)
Supplement: Supplementary file 1 — Additional file 1: Fig. S1. Some characteristics of CAR-M obtained through adenovirus infection. Fig. S2. Image of CAR-M phagocytosis of ovarian cancer cell. Fig. S3. Immunohistochemistry results of mice tumor tissue after HER2 CAR-M treatment. Fig. S4. Immunohistochemistry results of SKOV3 cell and HER2 CAR-M tumor bearing mice. Fig. S5. Immunohistochemistry results of SKOV3/A2780 cell and CD47 CAR-M tumor bearing mice. Fig. S6. Preliminary evaluation of the in vivo safety of CD47 CAR-M. [file 12967_2023_4479_MOESM1_ESM.docx]

**The application of HER2 and CD47 CAR-macrophage in ovarian cancer**

Additional Materials

Content

Figure S1. Page 2

Figure S2. Page 3

Figure S3. Page 4

Figure S4. Page 5

Figure S5. Page 6

Figure S6. Page 7


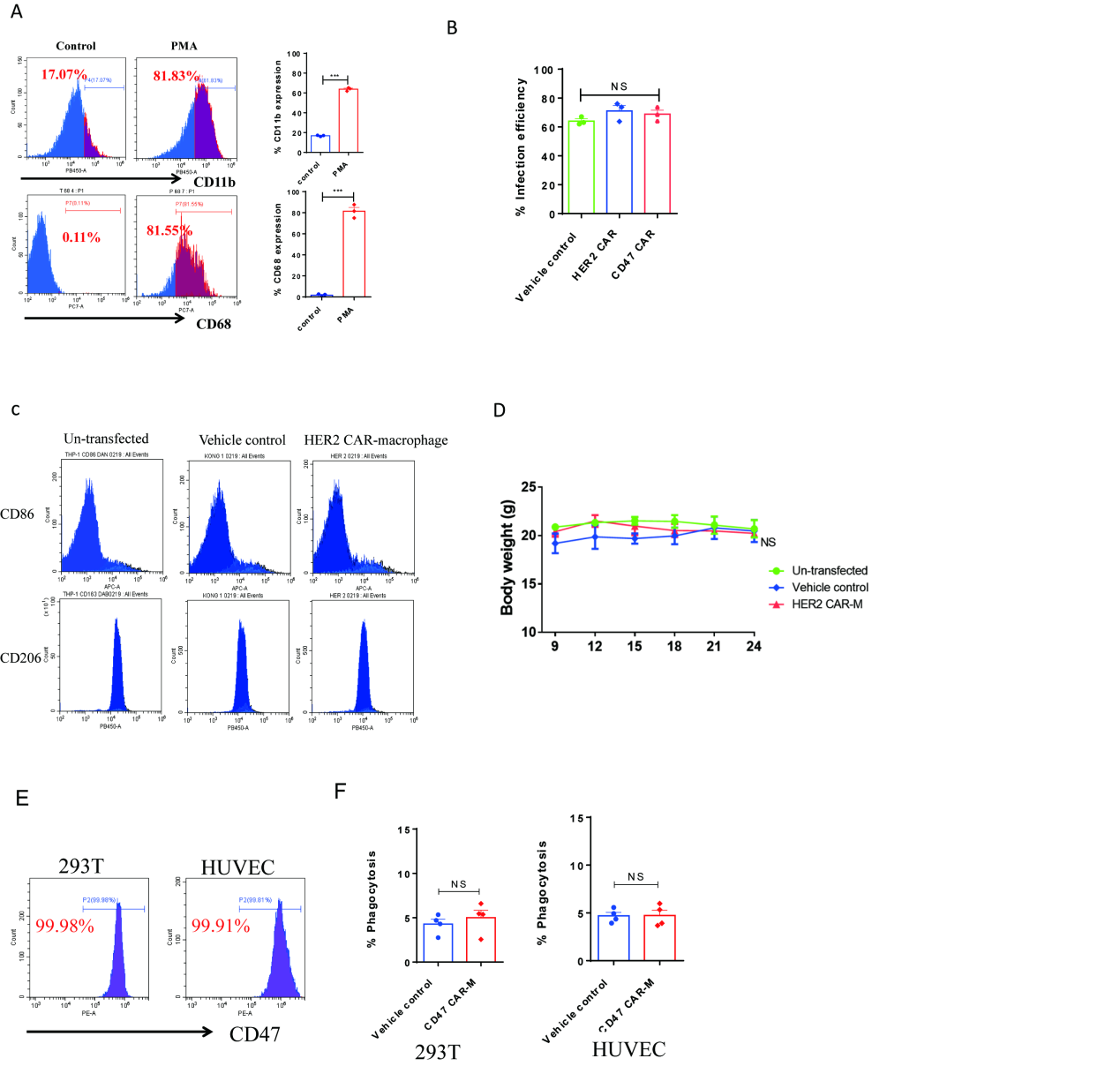


Fig S1. The PMA-induced differentiation efficiency of THP-1 cells to macrophage. (B) The infective efficiency of CAR adenovirus against THP-1-induced macrophages. (C) Phenotypic changes of macrophages after CAR adenovirus infection. (D) Body weight changes of mice after establishment of Hu-PBMC model. (E) The expression CD47 in 293T cells and HUVEC cells. (F) The phagocytosis of CD47 CAR-M on CD47hi 293T and CD47hi HUVEC (n=4). ns: not significant.


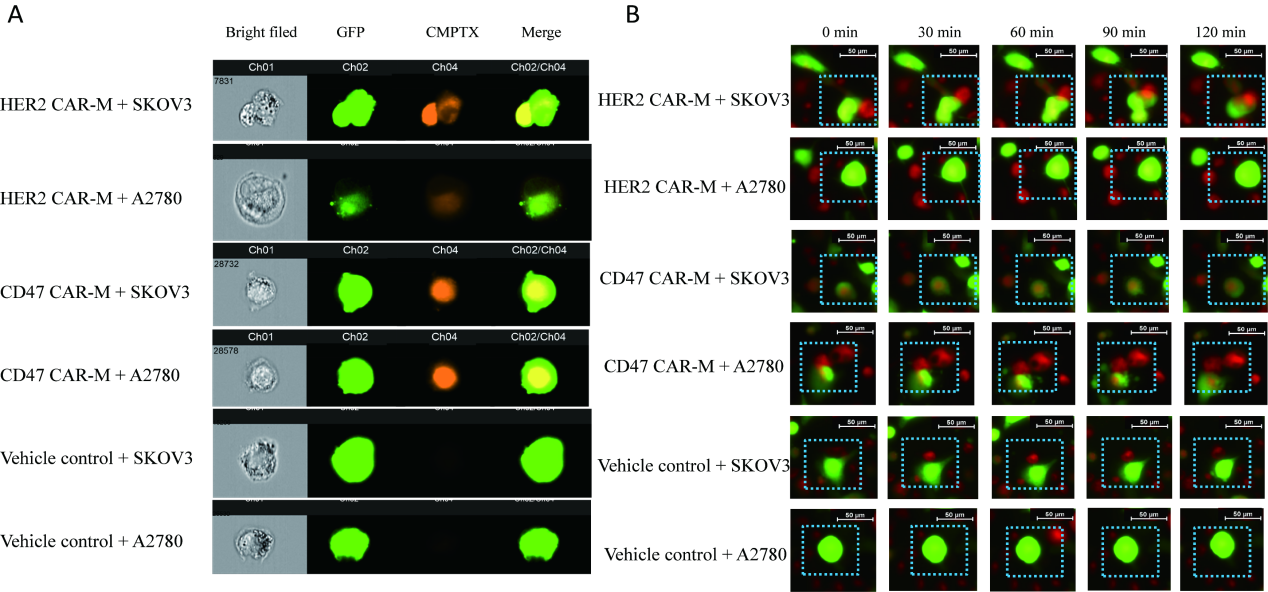


Fig S2. Image of CAR-macrophage phagocytosis of ovarian cancer cell. Imaging cytometry of Vehicle or HER2/CD47 CAR-macrophages after co-culture with SKOV3/A2780 target cells. Experiment was performed once. (B) Representative image of Live cell Imaging System of Vehicle or HER2/CD47 CAR-macrophages after co-culture with SKOV3/A2780 target cells. Experiment was performed once.


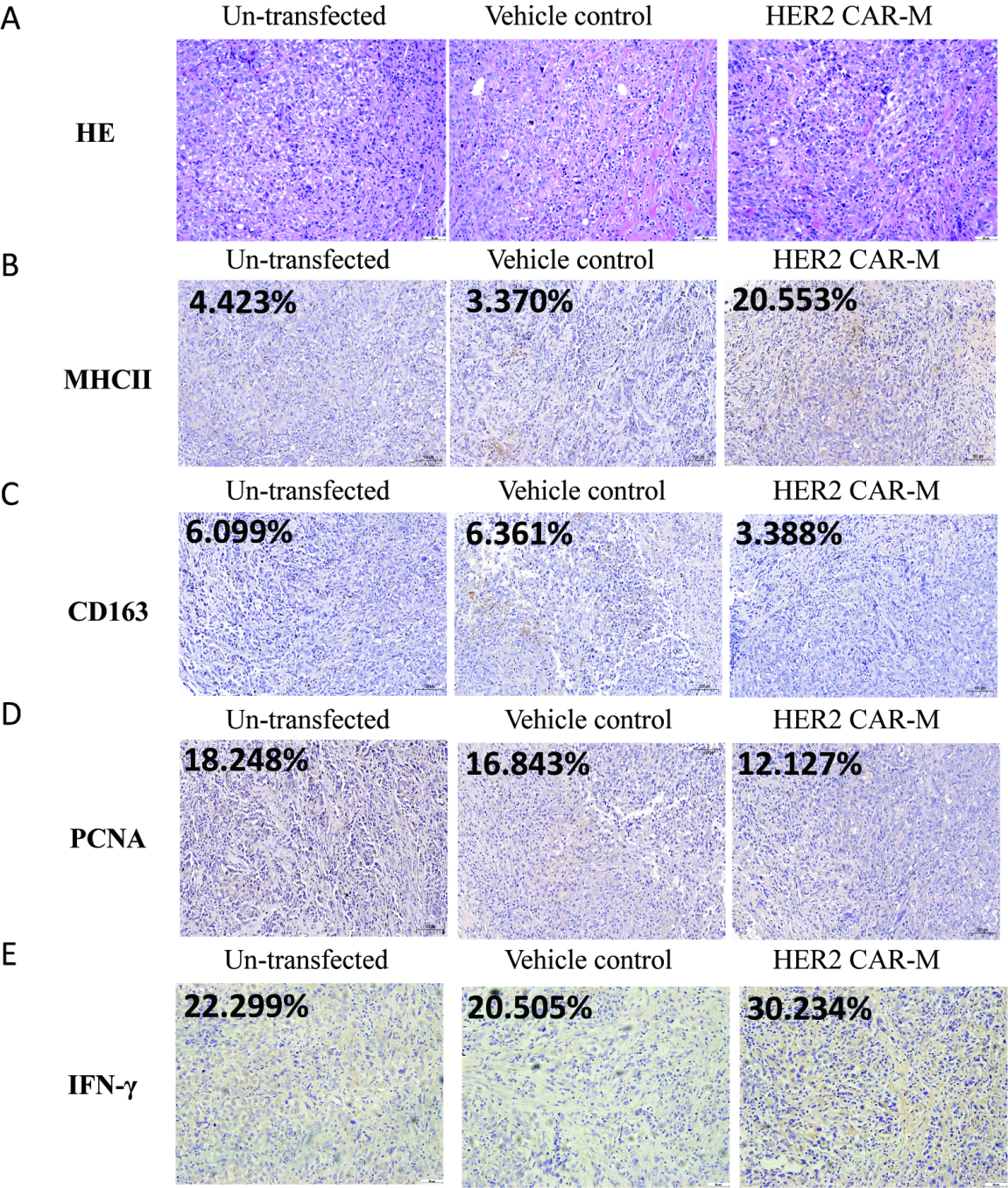


Fig S3. Immunohistochemistry results of mice tumor tissue after HER2 CAR-M treatment. Pathological HE staining of tumor tissue in NCG tumor-bearing mice after HER2-CAR-M treatment. (B) Immunohistochemical results of MHC II expression in tumor tissues. (C) Immunohistochemical results of CD163 expression in tumor tissues. (D) Immunohistochemical results of PCNA expression in tumor tissues. (E) Immunohistochemical results of IFN-γ expression in tumor tissues. A-E, 50μm.


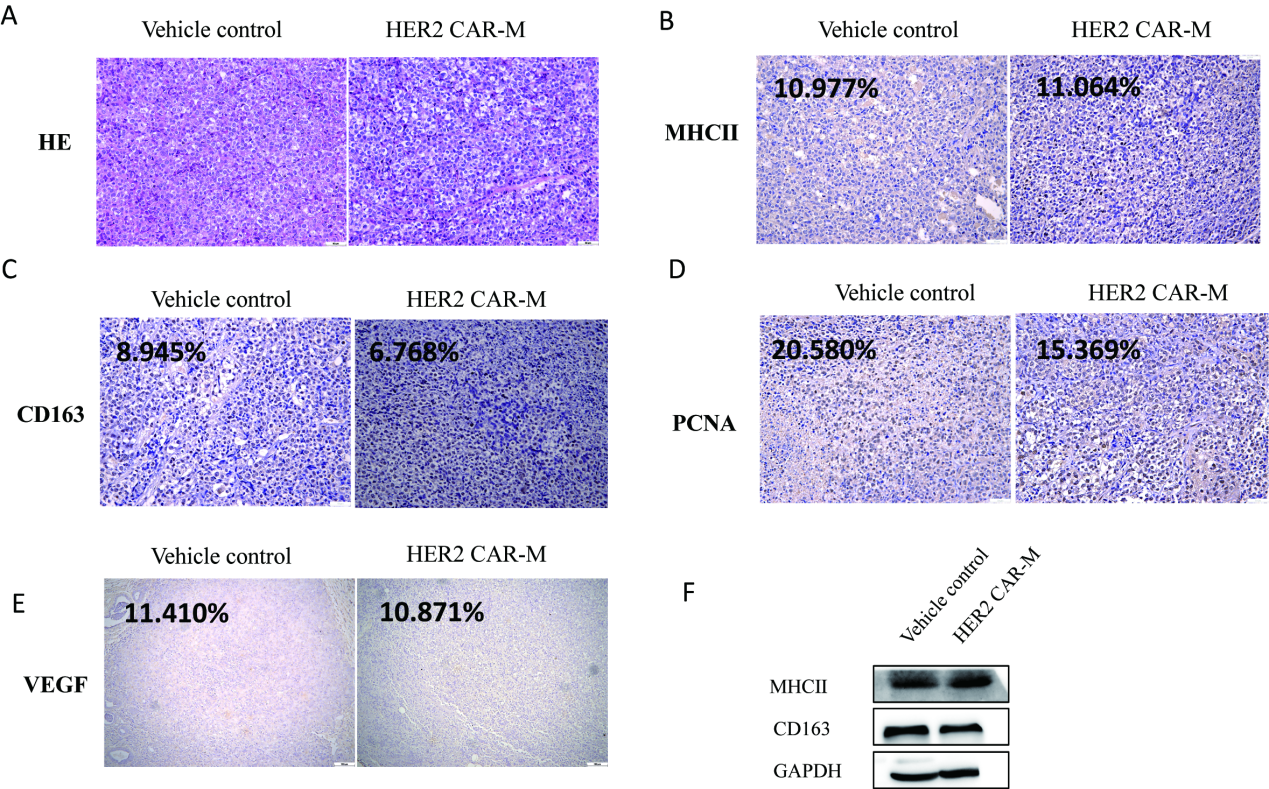


Fig S4. Immunohistochemistry results of SKOV3 cell and HER2 CAR-M tumor bearing mice. Pathological HE staining of tumor tissues (HER2 CAR-M and SKOV3). (B) Immunohistochemical results of MHCⅡ expression in tumor tissues. (C) Immunohistochemical results of CD163 expression in tumor tissues. (D) Immunohistochemical results of PCNA expression in tumor tissues. (E) Immunohistochemical results of VEGF expression in tumor tissues. (F) Western blot results of MHCII and CD163 expression in tumor tissues. A-D, 50μm; E, 100 μm.


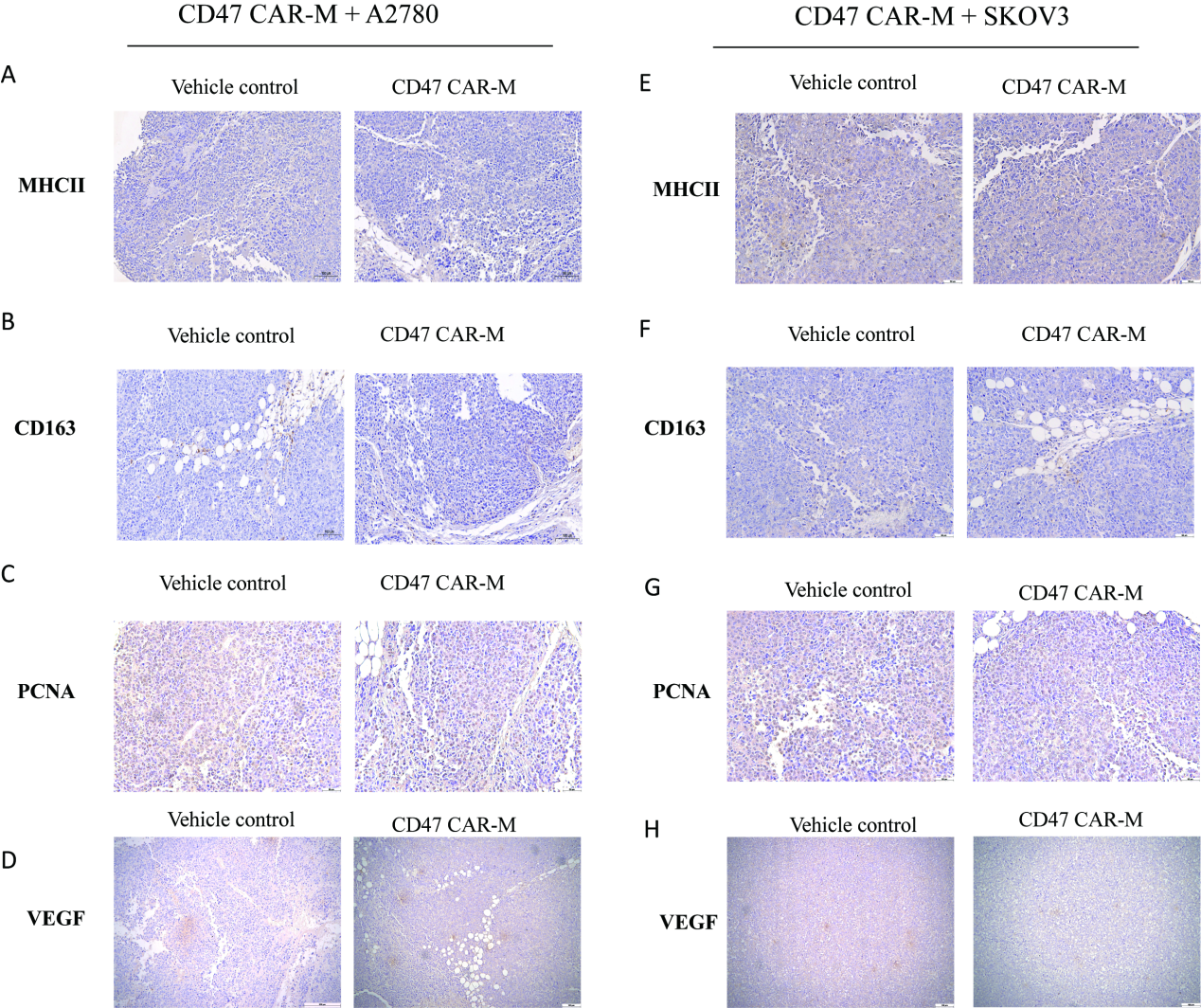


Fig S5. Immunohistochemistry results of SKOV3/A2780 cell and CD47 CAR-M tumor bearing mice. Immunohistochemical results of MHCⅡ expression in tumor tissues. (B) Immunohistochemical results of CD163 expression in tumor tissues. (C) Immunohistochemical results of PCNA expression in tumor tissues. (D) Immunohistochemical results of VEGF expression in tumor tissues. (E) Immunohistochemical results of MHCⅡ expression in tumor tissues. (F) Immunohistochemical results of CD163 expression in tumor tissues. (G) Immunohistochemical results of PCNA expression in tumor tissues. (H) Immunohistochemical results of VEGF expression in tumor tissues. A-C, E-G,50μm; D, H, 100μm.


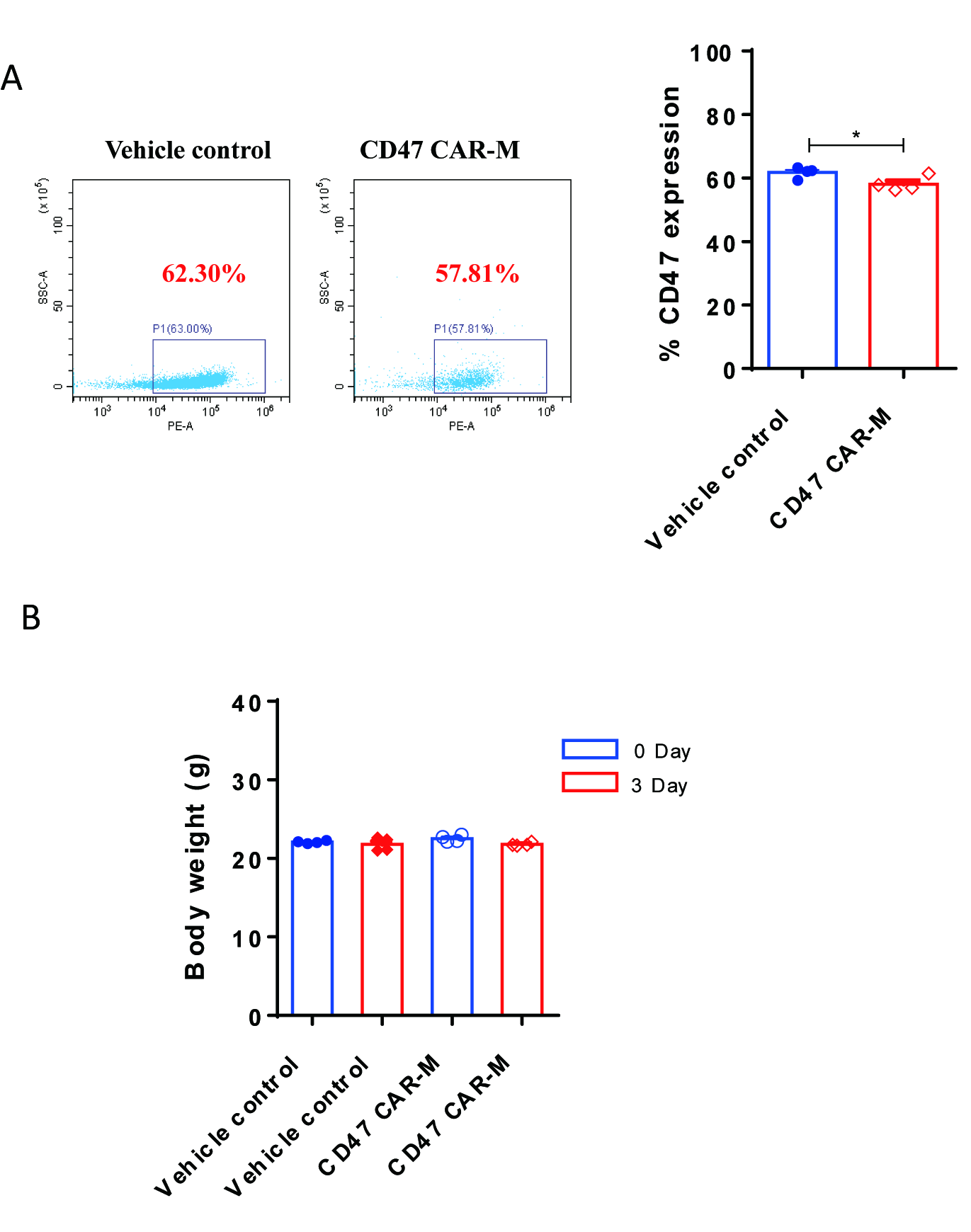


Fig S6. Preliminary evaluation of the vivo safety of CD47 CAR-M. The proportion of CD47^+^ erythrocyte in C57 mice treated with CD47 CAR-M. (B) Body weight changes in mice after tail vein injection of CD47 CAR-M. ns: not significant; ^*^ P<0.05.
